# Post-secondary classroom teaching quality evaluation using small object detection model

**DOI:** 10.1038/s41598-024-56505-4

**Published:** 2024-03-09

**Authors:** Rui Wang, Shaojie Chen, Gang Tian, Pengxiang Wang, Shi Ying

**Affiliations:** 1 Shangdong University of Science and Technology, Qingdao, 266590 China; 2 Wuhan University, Wuhan, 430072 China

**Keywords:** Information technology, Scientific data, Information technology, Scientific data

## Abstract

The classroom video has a complex background and dense targets. This study utilizes small object detection technology to analyze and evaluate students’ behavior in the classroom, aiming to objectively and accurately assess classroom quality. Firstly, noise is removed from the images using a median filter, and the contrast of the images is enhanced through histogram equalization. Label smoothing is applied to reduce the model’s sensitivity to labels. Then, features are extracted from the preprocessed images, and multi-scale feature fusion is employed to enhance semantic expression across multiple scales. Finally, a combination loss function is utilized to improve the accuracy of multi-object recognition tasks. Real-time detection of students’ behaviors in the classroom is performed based on the small object detection model. The average head-up rate in the classroom is calculated, and the quality of teaching is evaluated and analyzed. This study explores the methods and applications of small object detection technology based on actual teaching cases and analyzes and evaluates its effectiveness in evaluating the quality of higher education classroom teaching. The research findings demonstrate the significant importance of small object detection technology in effectively evaluating students’ learning conditions in higher education classrooms, leading to improved teaching quality and personalized education.

## Introduction

In the current context of increasingly fierce competition in post-secondary education, improving teaching quality and enhancing a university’s competitiveness have become important issues for every institution. The evaluation of classroom teaching quality is a crucial aspect of post-secondary education quality management and plays a significant role in improving teaching quality. It is of great importance to evaluate classroom teaching quality scientifically, objectively, comprehensively, and fairly in order to understand the performance of teachers, differentiate their qualities and teaching levels, motivate teachers’ enthusiasm for teaching, promote continuous improvement of teaching methods, enhance classroom teaching quality, and ensure the comprehensive development of talent cultivation goals.

Currently, there have been many discussions on the evaluation of classroom teaching quality in post-secondary education, with some based on theoretical frameworks and others based on practical applications. Zhu^1^ used factor analysis to statistically analyze sample data and identified significant factors that influence student satisfaction in classroom teaching. They established a classroom teaching effectiveness evaluation model based on student satisfaction. Chen and Su^2^ constructed a logistic model to explore the main factors affecting the quality of classroom teaching in post-secondary education from the perspectives of both students and teachers. Wang and Tan^3^ conducted a questionnaire survey of 1200 students from three local ordinary universities in Jiangxi Province to determine the importance and satisfaction level of various satisfaction factor indicators. They then created corresponding quadrant models to fully display the series of factors that contribute to student satisfaction, maintenance factors, opportunity factors, and improvement factors. Li and Zhao^4^ explored the satisfaction level of students regarding classroom teaching quality and identified influencing factors and weaknesses through exploratory factor analysis and structural equation modeling. Li and Jiang^5^ conducted statistical analysis on student evaluation data from 10 semesters in H University and concluded that student evaluations are primarily determined by the teaching quality of teachers, with limited impact on helping teachers improve their teaching abilities.

The evaluation methods mentioned above often rely on student questionnaires and self-evaluations by teachers, which may have issues such as random student responses and teachers overrating themselves. Therefore, there is a need to find a more objective, accurate, and authentic evaluation method. In recent years, the development of computer vision technology has provided new opportunities for education and teaching. Deep learning is a subfield of machine learning that has achieved significant success in computer vision tasks. By leveraging deep learning algorithms^6,7^, computer vision technology can effectively identify and localize target objects in images or videos. This technology has been widely applied in various detection tasks, such as industrial inspection^8^, face detection^9^, pedestrian detection^10^, vehicle detection^11^, medical detection^12^, and human action recognition^13^, achieving good results.

Meanwhile, some studies have explored methods for analyzing student engagement in the classroom based on computer vision. Parambil et al.^14^ utilized the YOLOv5 model to detect inattentive students during class and provide notifications to the teacher. Ngoc Anh et al.^15^ developed an automated system based on facial recognition technology to monitor students’ behaviors in the classroom. TS and Guddeti^16^ demonstrated a hybrid convolutional neural network for analyzing student body posture, gestures, and facial expressions to investigate engagement. Rashmi et al.^17^ proposed an automated system based on YOLOv3 to locate and recognize multiple actions of students within a single image frame. Wu et al.^18^ designed and developed a student classroom learning status feedback system that utilizes face pose recognition based on the YOLOv5s algorithm to quickly identify students’ learning status in the classroom. Huang et al.^19^ proposed a real-time multi-person student classroom behavior recognition algorithm based on deep spatio-temporal residual convolutional neural network. Wang et al.^20^ utilized the OpenPose algorithm to extract global features of human poses and combined them with the YOLOv3 algorithm to extract local features of interactive objects for the recognition and analysis of student behaviors.

Due to the characteristics of small targets in the classroom, such as low resolution, low contrast, and complex backgrounds, traditional object detection algorithms face difficulties in detecting small targets in the classroom. Small target detection techniques^21,22^ are an important research direction in computer vision aimed at addressing the problem of detecting and locating small objects in images or videos. Several optimization methods^23,24^ based on existing object detection algorithms have been proposed to reduce the cases of missed detection and false detection for small targets, thereby improving the detection performance of small targets. Research has shown that data augmentation^25,26^ can improve the detection performance of small targets by addressing the issues of low resolution, limited dataset quantity, and uneven distribution. To overcome the problem of information loss and unfavorable target localization caused by directly extracting features using convolutional neural networks for small targets, feature fusion^27–29^ can be performed on feature maps of different scales to overcome the bottleneck in feature extraction. Additionally, small targets may suffer from partial occlusion or high similarity with the surrounding background, leading to inaccurate or missed target localization. By using appropriate localization loss functions^30,31^, the object detection algorithm can be optimized to improve the localization accuracy of small targets. Similarly, small targets may exhibit unclear features or high similarity with other categories of targets, resulting in incorrect or confused classification. By utilizing suitable classification loss functions^32,33^, the object detection algorithm can be optimized to improve the classification accuracy of small targets.

Currently, there is relatively limited application of small object detection technology in classroom teaching quality evaluation. The utilization of improved small object detection technology has great potential to enhance the evaluation of teaching quality in classrooms. This paper proposes a classroom teaching quality evaluation method based on the small object detection model YOLOv5. By collecting a large number of video samples from classroom teaching, the method trains and improves the model to achieve high accuracy real-time recognition and calculation of students’ head-up rate. By continuously monitoring the head-up rate in the classroom, it can objectively evaluate the teaching quality, breaking away from the subjective and random scoring methods currently used in universities. By introducing computer vision technology, this scientific approach to evaluating classroom teaching quality can be applied in the field of post-secondary education, potentially driving innovative development in post-secondary education and contributing to the cultivation of high-quality talents to meet future demands.

The main contributions of our paper are as follows:

1. We propose a novel method for assessing classroom teaching quality based on small object detection technology. By utilizing the YOLOv5 model, we are able to achieve real-time recognition and calculation of students’ head-up rate, providing an objective and accurate measure of teaching effectiveness.
2. We demonstrate the potential of small object detection technology in the field of education by applying it to the evaluation of classroom teaching quality. This application expands the use of computer vision technology and provides a scientific approach to assess teaching quality, moving away from subjective and random scoring methods currently used in universities.

The main structure of this paper: “Introduction” section introduces the background. “Classroom real-time head-up rate detection model based on small object detection” section introduces the method framework and details our approach. “Classroom teaching quality evaluation based on real-time head-up rate detection model” section is our experimental scheme and result analysis. “Conclusion draws conclusions and summarizes the next steps.

## Classroom real-time head-up rate detection model based on small object detection

The target object for classroom head-up detection is the face, which falls under small object recognition. YOLOv5 (You Only Look Once)^34,35^, as a deep learning-based object detection model, can be used to detect and recognize small objects in complex classroom video backgrounds with dense targets. The specific steps include data preprocessing, feature extraction, and object detection.

### Data preprocessing

#### Median filtering

Due to interference from environmental lighting and signal transmission, there is noise present in the video images of the classroom. This noise reduces image clarity and blurs the boundaries of the target objects, making feature extraction and recognition tasks difficult. Therefore, it is necessary to remove the noise from the images, and one commonly used denoising method is the median filter. The median filter works by sorting the pixel values in the neighborhood of each pixel and replacing the pixel value with the median value. This helps to eliminate isolated noise points. The operation of the median filter can be represented as follows:

1 $$\begin{aligned} I_{denoised} (x,y)=median(I_{original} (x+i,y+j)). \end{aligned}$$

In Eq. (1), $I_{denoised} (x,y)$ represents the pixel value of the denoised pixel at position (*x*, *y*), and $I_{original} (x+i,y+j)$ represents the value of the pixel $(x+i,y+j)$ in the original image. The variables (*i*, *j*) represent the size and position of the filter. By applying the median filter to the original video image dataset, a new image dataset is generated.

#### Histogram equalization

In the classroom video, small objects often have low contrast, which makes them similar to the background and reduces the visibility of edge and texture details. This makes it difficult to accurately recognize and segment small objects, which affects feature extraction and recognition tasks. By enhancing the contrast of the image, the edges and texture details can be highlighted, which helps in the delineation of the boundary between the target object and the background. One commonly used method for contrast enhancement is histogram equalization. Histogram equalization adjusts the pixel value distribution of an image to make it evenly distributed throughout the entire range, thereby enhancing the contrast of the image. The operation of histogram equalization can be represented as:

2 $$\begin{aligned} I_{enhanced} (x,y)= \frac{CDF(I_{denoised} (x,y))-\min(CDF)}{(N\times M)-\min(CDF)} \times (\max(CDF)-\min(CDF)). \end{aligned}$$

In Eq. (2), $I_{enhanced} (x,y)$ represents the enhanced image pixel value, $CDF(I_{denoised} (x,y))$ represents the cumulative distribution function of the image pixel value, $N\times M$ represents the total number of pixels in the image, and *max*(*CDF*) and *min*(*CDF*) represent the maximum and minimum values of the cumulative distribution function, respectively. Histogram equalization is used to remap the grayscale level of the filtered image dataset, highlighting the edge information of the human body, which helps in the object segmentation task in the classroom video.

#### Label smoothing

Classroom video images enhanced with contrast contain feature information but lack semantic information, making them unsuitable for direct image categorization. Therefore, image annotation is required to overlay semantic information. In object detection tasks, bounding boxes can be used in the images to frame the target objects and add text information indicating their coordinates, sizes, and types. In traditional object detection models, each target is labeled with a binary value indicating its presence or absence. The model is optimized based on these labels to enable correct classification of input data during testing. However, the annotations in training data may have accuracy issues, and incorrect annotations can negatively impact the model’s learning process. When the model overly focuses on erroneous label information, it can lead to overfitting and excessive reliance on label information, which reduces the model’s generalization ability in similar tasks and affects its performance. To address this issue, label smoothing can be used to reduce the model’s sensitivity to labels. In the YOLOv5 model, a smoothing coefficient is set to allocate a portion of the true label’s confidence to the background label. This allows the model to focus more on the input data’s features during training and reduce its dependence on label information. Label smoothing enhances the model’s robustness, reduces the risk of overfitting, and makes the model more stable and reliable. Mathematically, label smoothing can be represented as:

3 $$\begin{aligned} smooth_{label}=(1-\sigma ) \times true_{label} + \sigma \times background_{label}. \end{aligned}$$

In Eq. (3), $smooth_{label}$ represents the smoothed label, $true_{label}$ represents the presence or absence of the target, usually denoted as 1 or 0, and $background_{label}$ represents the presence or absence of the background, also typically denoted as 1 or 0. $\sigma$ is a smoothing parameter ranging between 0 and 1, used to control the degree of smoothing. When $\sigma$ approaches 0, it indicates no smoothing, preserving the original binary labels, and the model focuses more on the input data’s features. On the other hand, when $\sigma$ approaches 1, it indicates complete smoothing, where the background label receives more confidence, and the model pays more attention to the input data’s feature information. By using label smoothing, even in cases with imperfect manual annotation, the recognition accuracy can still be ensured, reducing the impact of imprecise anchor box positioning or annotation errors on the model’s accuracy.

### Feature extraction and fusion

#### Feature extraction and importance analysis

Due to the complexity of information contained in classroom video images, it is not conducive to the processing and analysis of subsequent object detection tasks. Therefore, feature extraction is required for preprocessed images, which involves converting the information in the images into numeric feature vectors.

**Figure 1:**
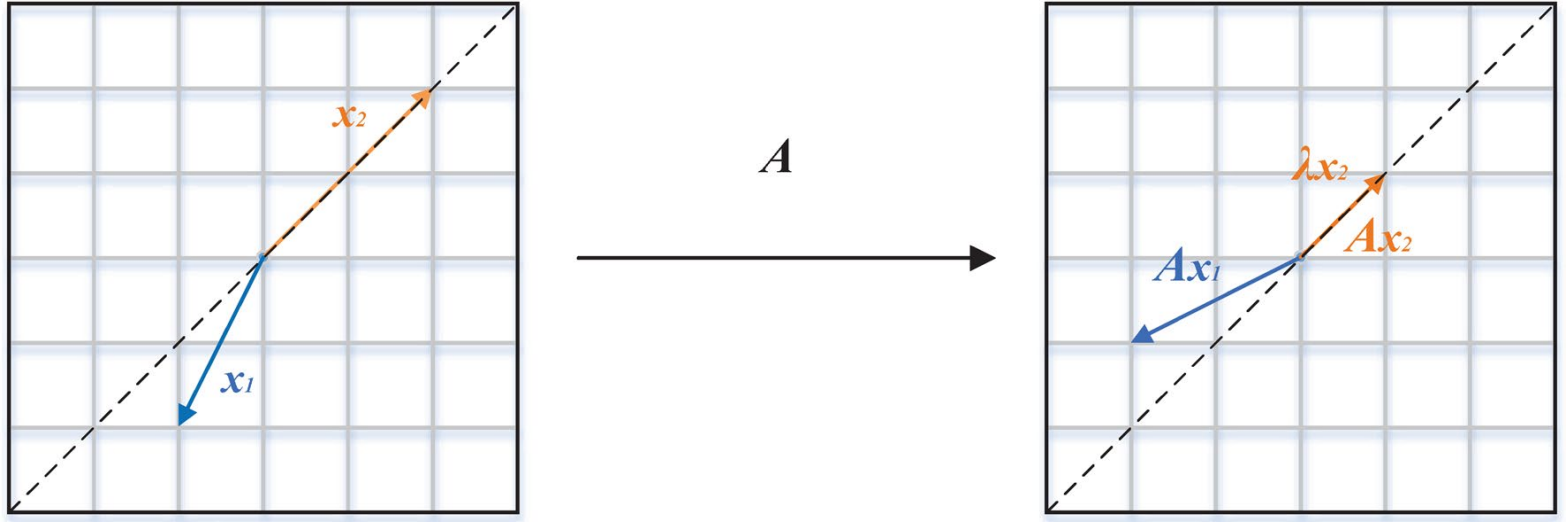
Matrix mapping.

An image can be represented as a matrix composed of pixels, which contains numerous vectors. Matrix multiplication represents a mapping from one vector space to another vector space. As shown in Fig. 1, $x_1$ is mapped to $A x_1$ after matrix multiplication, and $x_2$ is mapped to $A x_2$. The vector $x_1$ before mapping and its corresponding mapped vector $A x_1$ are not on the same line, and $x_2$ and its corresponding mapped vector $A x_2$ are not on the same line either. Vectors that lie on the same line before and after mapping are called eigenvectors. The matrix only scales and changes these types of vectors. Therefore, the vector $A x_2$ can be written as $\lambda x_2$, where $\lambda$ is the eigenvalue of the matrix *A*. A invertible matrix can be decomposed into a product of eigenvalues and eigenvectors.

4 $$\begin{aligned} Bx=\lambda x \\ (B-\lambda I)x=0 \\ |B-\lambda I|=0. \end{aligned}$$

In Eq. (4), where *B* is an arbitrary matrix and *I* is the identity matrix, the eigenvalues and eigenvectors can be calculated. The eigenvalue represents the strength of the corresponding eigenvector. By converting abstract features into numerical feature vectors, the eigenvalues correspond to the amount of information contained in the direction of the eigenvectors. The ratio of a specific eigenvalue to the sum of all eigenvalues represents the variance contribution rate of the corresponding eigenvector. The data transformed by the eigenvector transformation is called the principal component of the variables. When the cumulative variance contribution rate of the current m principal components reaches a high proportion, these *m* components can be retained, achieving dimensionality reduction of the image data. Dimensionality reduction reduces the dimensionality of the data while retaining the most representative and important features, thus improving the efficiency of subsequent processing and analysis.

#### Multiscale feature fusion

The target object in the classroom head detection task is the face, which is considered a small object in the recognition task. Object recognition requires extracting more abstract deep features through multiple convolutional layers. However, when the convolutional kernel slides over the input data, it reduces the spatial dimensions of the input data, leading to a decrease in the size of the feature maps and loss of feature information for small objects. In order to accurately detect small objects, one approach is to use image pyramids of different scales for the same image. However, processing multi-scale images requires large memory and training time. To address this issue, feature pyramids can be incorporated into the object detection process. Specifically, the network structure called Feature Pyramid Network (FPN) can be used. FPN upsamples the upper-level feature maps to obtain new feature maps and then fuses them with the same-level feature maps. This allows the transfer of deep semantic features to shallow layers and enhances semantic representation capabilities across multiple scales. FPN is typically positioned after the backbone network in the network architecture.

5 $$\begin{aligned} P_n=Conv(P_{n-1} )+P_{n-1}\\ P_n'=Conv(P_n )+P_n\\ P_n'=Conv_{1\times 1} (UpS P_{n-1})\\ P_n^{new}=Conv_{3\times 3} (P_n+P_n'). \end{aligned}$$

In the FPN structure, $P_{n-1}$ represents the input feature map from the previous layer, $P_n$ represents the output feature map of the current layer, *Conv* represents the convolution operation. $P_n'$ represents the new feature map constructed based on the original feature map of the $(n-1)$-th layer, $Conv_{*\times *}$ represents the convolution operation with a kernel size of $*\times *$, UpS represents the upsampling operation, $P_{n-1}$ represents the original feature map of the $(n-1)$-th layer, and $P_n^{new}$ represents the fused feature map of the *n*-th layer.

In the spatial dimensions (width and height), an upsampling operation is performed to ensure that the width and height of the original upper-level feature map and the new feature map are consistent. Then, a $1\times 1$ convolution is applied to unify the depth (number of channels). The new feature map is element-wise added to the corresponding elements in the original lower-level feature map, achieving the fusion of the upper-level and lower-level features. To address the potential issue of insufficient fusion caused by direct addition of corresponding elements in the two feature maps, FPN applies a $3\times 3$ convolution for smoothing on the fused feature map. This results in a more comprehensive fused feature map.

Small objects in classroom videos, such as students’ faces, often have limited feature information due to their small size, which poses challenges for accurate detection and recognition by models. To address this issue, this study utilizes the Feature Pyramid Network (FPN), which introduces multiple scales of feature maps and performs feature fusion through upsampling and convolution operations, allowing integration of semantic information from different scales even in the shallow layers of the network. This process includes preprocessing of the images through noise removal and contrast enhancement. Then, features are extracted using the FPN architecture and feature fusion is performed through upsampling and convolution operations to achieve semantic expression across multiple scales. This approach ensures that the model can access comprehensive semantic information about small objects, even in the shallow layers. Through multi-scale feature fusion, our model can more accurately detect and locate students’ behaviors in the classroom, thereby facilitating the evaluation and analysis of classroom quality.

### Object detection

The task of object detection is to identify specific objects from an image or video sequence and determine their category and location, separating them from the background. The tasks of object detection include: detecting the objects in the image or video sequence, classifying the objects, and determining their position and size. In the YOLOv5 model, features are extracted to make predictions. The model predicts the position and classification of the objects using predicted bounding boxes and corresponding class labels. By regressing the gradients of the loss function, the model improves the classification, localization, and confidence of the predictions. Non-maximum suppression is applied to ensure the uniqueness of the predicted bounding boxes for the objects.

#### Loss function

In a complex classroom video background with numerous and densely packed targets, and varying distances between the targets and the camera, the sizes of the targets can vary significantly. The loss function reflects the difference between the predicted bounding boxes and the ground truth boxes, and through gradient backpropagation, the predicted boxes continuously approach the ground truth boxes. However, different loss functions have different abilities to reflect the differences between the predicted boxes and the ground truth boxes. A good loss function can better reflect these differences and achieve better gradient backpropagation, resulting in more accurate localization and improved prediction accuracy. YOLOv5 uses a combination of loss functions, which mainly includes three parts: localization loss, confidence loss, and classification loss.

##### Localization loss

The mean squared error (MSE) loss function is used to measure the difference between predicted bounding boxes and ground truth bounding boxes, which reflects the accuracy of the model in predicting the position of the target bounding boxes. The calculation of MSE is as follows:

6 $$\begin{aligned} L_{CIoU} (b,b_{gt})=1-CIoU=1-(IoU-\frac{\rho ^2 (b,b_{gt})}{c^2} - \frac{v^2}{1-IoU+v}). \end{aligned}$$

$L_{CIoU}$ stands for Localization Consistency IoU, where IoU refers to the Intersection over Union of the predicted box and the ground truth box. $c^2$ represents the square of the diagonal length of the minimum bounding rectangle of the predicted box and the ground truth box. $\rho ^2 (b,b_{gt})$ represents the square of the distance between the center points of the predicted box and the ground truth box. $v= \frac{4}{\pi ^2} (arctan \frac{w^{gt}}{h^{gt}} -arctan \frac{b_w}{h_w})^2$, it is used to measure the similarity of aspect ratios, where arctan $\frac{w^{gt}}{h^{gt}}$ represents the aspect ratio of the ground truth box and arctan $\frac{b_w}{h_w}$ represents the aspect ratio of the predicted box.

##### Classification loss

The class loss for predicting targets is calculated based on the class scores of the predicted bounding boxes and the classification values of the target boxes. In YOLOv5, the cross-entropy loss function is used to measure the difference between the predicted class and the true class, which evaluates the model’s accuracy in recognizing the target class. The calculation method is as follows:

7 $$\begin{aligned}{} & {} BCEWithLogitLoSS=\frac{1}{N} \sum _{i=1}^N (y_i \cdot log(\sigma (p_i))+(1-y_i) \cdot log(1-\sigma (p_i))), \end{aligned}$$

8 $$\begin{aligned}{} & {} \sigma (x_i)=Sigmiod(x_i)=\frac{1}{1+e^{-x_i}}. \end{aligned}$$

Whereas, *N* represents the total number of samples, log is the natural logarithm, $p_i$ is the probability of sample $x_i$ being predicted as positive, and $y_i$ is the true label of sample $x_i$. In binary classification problems, $y_i$ takes a value of either 1 or 0, indicating whether sample $x_i$ belongs to the positive class or not.

9 $$\begin{aligned} L_{cls} (c_p,c_{gt})=BCE_{cls}^{sig} (c_p,c_{gt}; w_{cls}). \end{aligned}$$

Whereas, $L_{cls}$ represents the class loss, $BCE^{sig}$ refers to the binary cross-entropy function *BCEWithLogitsLoss* with *Sigmoid*, $c_p$ and $c_{gt}$ respectively denote the predicted box class score and the target box class categorical value, and $w_{cls}$ is the weight for the classification loss.

##### Confidence loss

Confidence loss for predicting targets refers to the model’s confidence level in the existence of the targets. The confidence loss for targets is calculated using sample pairs obtained from positive sample matching. The calculation method is as follows:

10 $$\begin{aligned} L_{obj} (p_o,p_{IoU})=BCE_{obj}^{sig} (p_o,p_{IoU}; w_{obj}), \end{aligned}$$

where $L_{obj}$ is the confidence loss, $p_o$ and $p_{IoU}$ represent the confidence score and IoU value of the predicted bounding box, and $w_{obj}$ is the weight for the confidence loss.

YOLOv5 utilizes a combined loss function to improve the accuracy of object detection in classroom video recognition. By minimizing the localization loss, it ensures precise localization of the target object and enhances the accuracy of object detection. Minimizing the classification loss allows the model to accurately determine the category of the target object, further improving object detection accuracy. The confidence loss helps reduce false detections by evaluating the presence of the target object. By combining these losses, YOLOv5 comprehensively considers the position, category, and presence of target objects, resulting in improved overall accuracy in object detection tasks. This comprehensive loss function effectively addresses the challenge of detecting objects of different sizes in classroom video analysis. For instance, it can detect and analyze small objects such as students raising or lowering their heads. This enables a precise understanding of students’ learning conditions.

#### Non-maximum suppression

In the task of object detection in classroom videos, where there are numerous and densely-packed students, it is common to have multiple bounding boxes covering the same student. To avoid counting the same student multiple times, it is necessary to filter out redundant bounding boxes and keep only the one with the highest confidence score. This process is known as non-maximum suppression (NMS). Here is a specific implementation scheme for NMS.

- *Step 1* Sort the bounding boxes based on their confidence scores in descending order.
- *Step 2* Select the bounding box with the highest confidence score (referred to as the current best box) and add it to the output list, removing it from the list of remaining bounding boxes.
- *Step 3* Calculate the Intersection over Union (IoU) between the current best box and the remaining bounding boxes.
- *Step 4* For the bounding boxes with an IoU greater than a predefined threshold with the current best box, consider them to represent the same student and suppress them by removing them from the list of remaining bounding boxes.
- *Step 5* Repeat steps 2 to 4 until there are no remaining bounding boxes.

By applying non-maximum suppression, we can ensure that each student is counted only once, resolving the issue of multiple counts. In the context of object detection in classroom videos, this approach prevents the number of bounding boxes from exceeding the number of actual students. By accurately detecting the position of each student through NMS, we can maintain the accuracy of subsequent calculations, such as computing the rate of students paying attention in the classroom.

### Classroom real-time head-up rate detection

Based on the object detection model, the selected classroom video data is processed frame by frame to detect and classify whether students are raising their heads or not. For each frame, the system determines the head posture of each student, such as whether the head is raised or lowered. This allows for the determination of the head-up situation of students in each frame. The system records in real-time the head-down and head-up situations of each student and calculates the average head-up rate in the entire classroom. This method can evaluate the head-up situation in the classroom in real time and accurately, providing data support for classroom teaching quality evaluation.

#### Classroom real-time head-up rate

The classroom real-time head-up rate refers to the ratio of the number of students looking up at a certain point in time to the total number of students in the class, which can reflect the overall head-up situation of the students at that particular time.

#### Classroom average head-up rate

Classroom head-up rate refers to the ratio of the number of students looking up to the total number of students detected in a class, which can reflect the overall head-up situation of the students during the entire class. The average classroom head-up rate can be used as an important indicator for evaluating the quality of classroom teaching. By calculating the ratio of the number of students detected looking up to the total number of students in the class, summing up the results, and calculating the average value, the average overall head-up rate of the class can be obtained. The formula for calculating the average classroom head-up rate is: $\beta =\frac{1}{X} \sum _{x=1}^X \alpha _x$, where *X* is the number of times the head-up rate is detected ($\geq 5$ times, calculated every 5 min), and $\alpha _x$ is the real-time head-up rate.

## Classroom teaching quality evaluation based on real-time head-up rate detection model

Based on real classroom teaching cases, this study utilizes small object detection technology to analyze and evaluate the learning status of students in the classroom. By installing cameras in the classroom, behavior data of students is collected and processed through data preprocessing and feature extraction. The small object detection algorithm is then employed to identify and analyze the students’ learning status. Subsequently, based on evaluation indicators and standards, the teaching effectiveness of the teacher and the quality of the classroom are evaluated and analyzed.

### Method procedure

**Figure 2:**
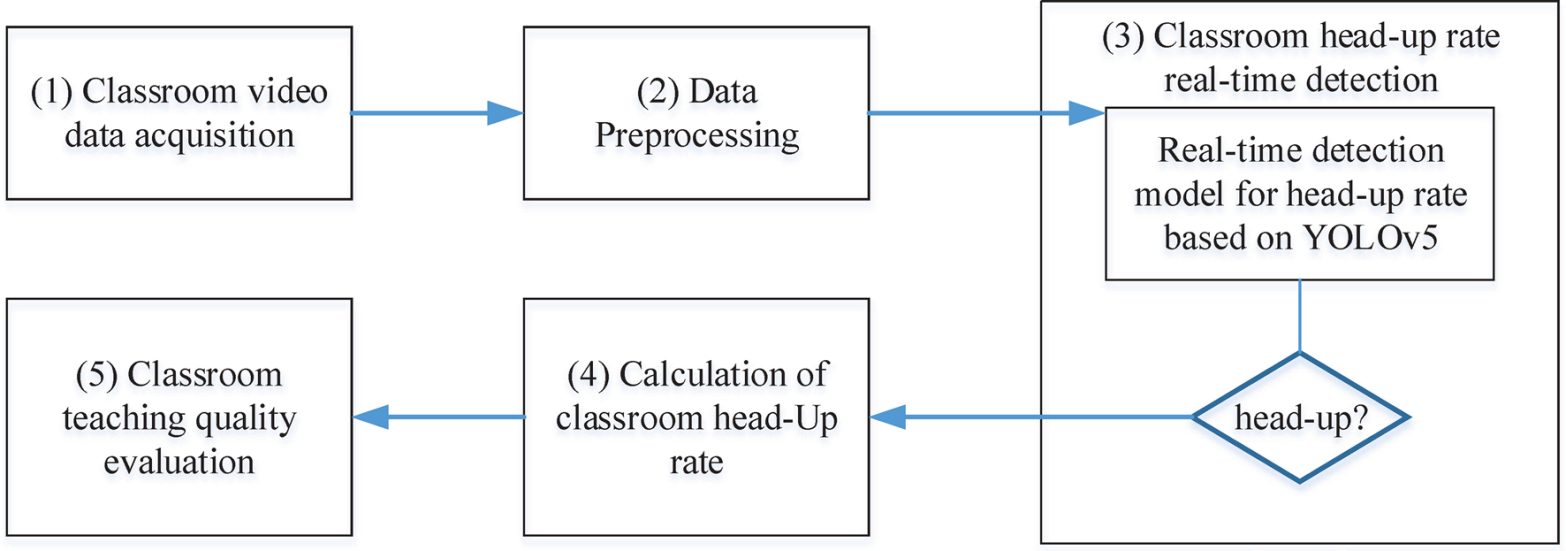
Classroom teaching quality evaluation procedure based on real-time head-up rate detection model.

The procedure of classroom teaching quality evaluation method based on real-time head-up rate detection model is shown in Fig. 2. It mainly includes:

1. *Classroom video data acquisition*. Obtain student video data monitored by cameras in the classroom through the school monitoring system.
2. *Data Preprocessing*. The video data is preprocessed to provide better input for the real-time head-up rate detection model in the classroom. This includes image enhancement techniques to improve the robustness and diversity of the model. Additionally, the student’s posture is labeled through data annotation, indicating whether they are raising their heads or not.
3. *Classroom real-time head-up rate detection*. The preprocessed data is inputted into a real-time detection model based on YOLOv5. The model evaluates the posture of each student and determines whether they are raising their heads or not.
4. *Calculation of classroom head-up rate*. The number of students raising their heads and the total number of students in the classroom are counted. This information is used to calculate the real-time head-up rate and the average head-up rate in the classroom.
5. *Classroom teaching quality evaluation*. Based on the calculation results of the head-up rate in the classroom, we can explain the feedback effect of the students’ head-up rate on their participation and attentiveness in the class. A higher head-up rate indicates that students are more focused and actively engaged in the classroom activities, reflecting a higher quality of teaching. Conversely, a lower head-up rate suggests that students are distracted or uninterested in the teaching content, indicating a need for improvement in the teaching quality. Therefore, the head-up rate can be used as an indicator to assess the level of student participation and attentiveness in the classroom, thereby evaluating the quality of classroom teaching. Based on specific research and practice, the classroom with the highest head-up rate is taken as the standard, and the head-up rates of other classrooms are normalized accordingly. Based on this, the evaluation results of post-secondary classroom teaching quality based on real-time head-up rate detection are divided into five levels, as shown in Table 1.

**Table 1:** Evaluation levels of post-secondary classroom teaching quality based on real-time head-up rate detection.

Level	Standards	
	Normalized head-up rate	Description
Excellent	Above 90%	Students demonstrate a very high level of focus and engagement in the classroom, with a strong understanding and grasp of the content
Good	Between 80 and 90%	Students exhibit a high level of focus and engagement in the classroom, with a good understanding and grasp of the content
Average	Between 70 and 80%	Students display a moderate level of focus and engagement in the classroom, with some understanding and grasp of the content
Fair	Between 60 and 70%	Students display a moderate level of focus and engagement in the classroom, with some understanding and grasp of the content
Poor	Below 60%	Students display a moderate level of focus and engagement in the classroom, with some understanding and grasp of the content

### Classroom teaching quality evaluation based on real-time head-up rate detection model

#### Classroom video data acquisition

The data was obtained from the Dean’s Office of Shandong University of Science and Technology. Specifically, 100 classroom videos from the university’s 2022-2023-2 academic semester were collected through the classroom monitoring system. These videos were used to build an accurate real-time detection model for classroom head pose. To ensure the effectiveness of model training and evaluation, the classroom videos were divided into frames at a predetermined frame interval. These frames were then extracted and used as part of the dataset for this study.

#### Data preprocessing

**Figure 3:**
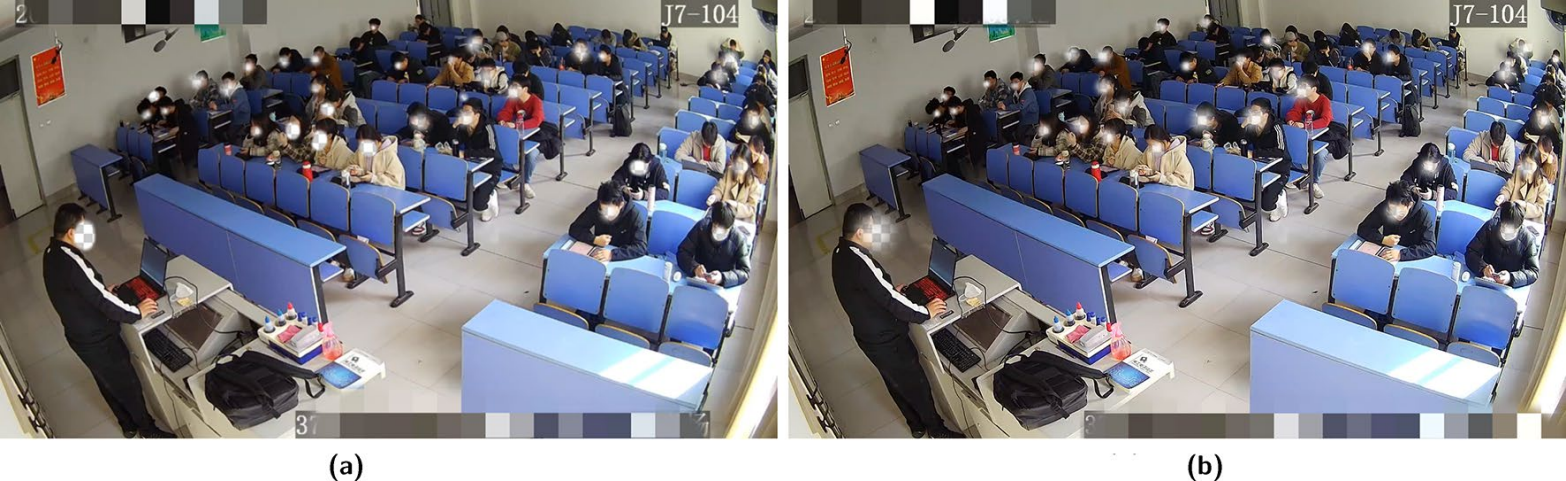
Frame image enhancement in the classroom video.

To increase the diversity and richness of the data samples, the Mosaic data augmentation technique^36^ was used to concatenate four randomly cropped sub-images into a new image. This makes the dataset more representative and improves the model’s generalization ability. To enhance the clarity of the classroom video images, an interactive super-resolution modulation method based on metric learning^37^ was employed. This enhances the details and clarity of the images, improving the accuracy of the model in detecting head poses. The before and after comparison of frame image enhancement in the classroom video is shown in Fig. 3.

#### Classroom real-time head-up rate detection

**Figure 4:**
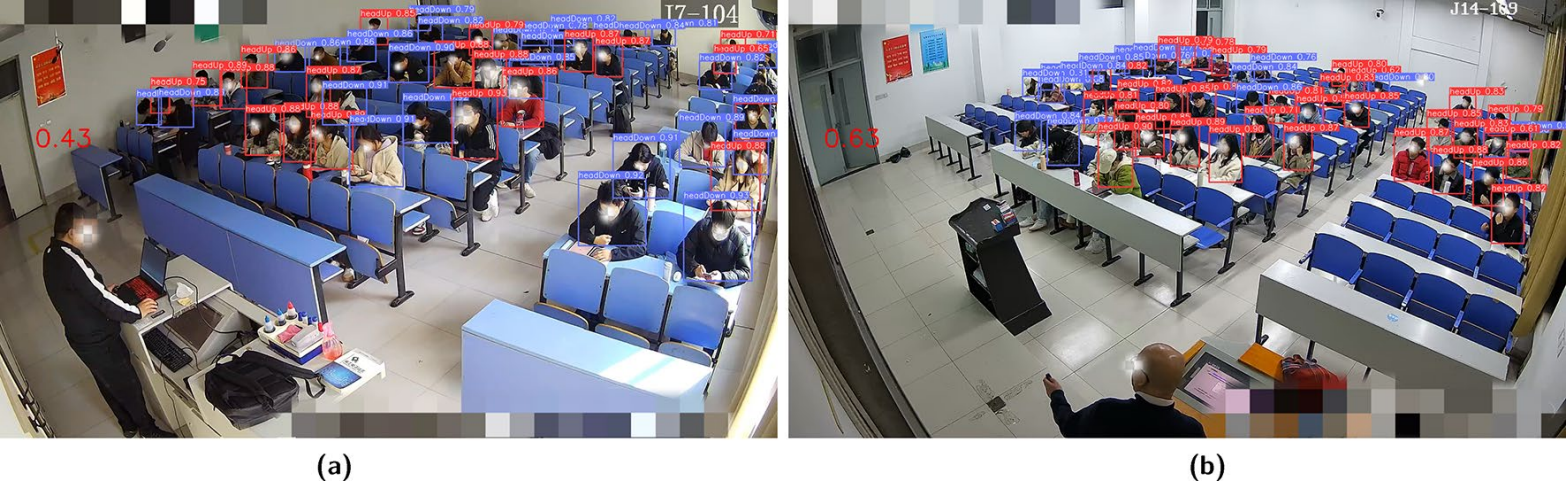
Real-time detection results of average classroom head pose rate.

To train the model, the labelImg image annotation tool was used to label the head poses of students in the classroom. The student head images were annotated as “0” for head-up and “1” for head-down. A total of 800 student head images were annotated. To evaluate the performance of the model, the dataset was divided into a training set (800 images), a validation set (100 images), and a test set (100 images). The real-time detection model for classroom head pose was trained, and it was used to determine the head pose of each student, whether they are looking up or not, as shown in Fig. 4.

#### Model training

**Figure 5:**
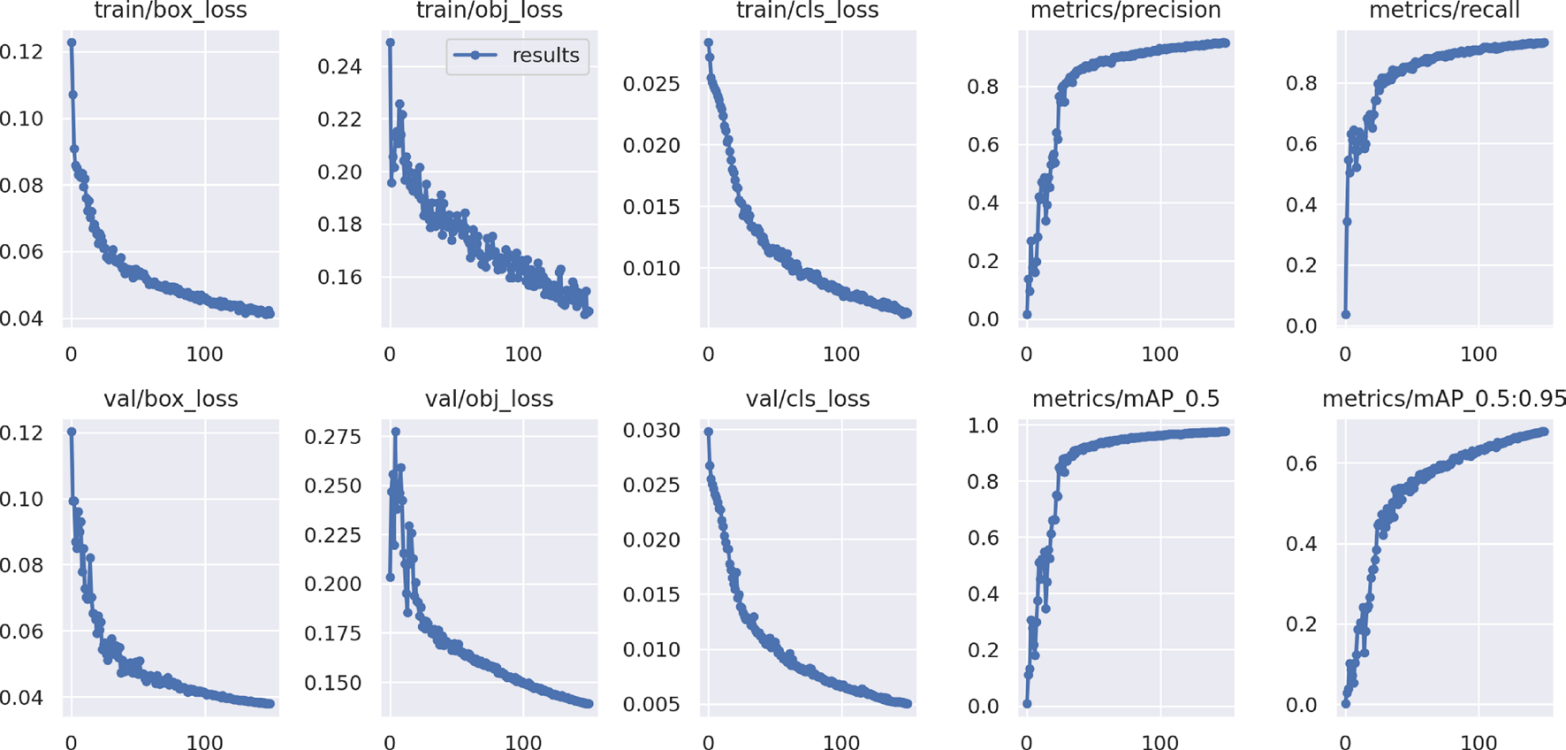
Training results.

The main parameter settings during training were as follows: the input image size was set to 640 $\times$ 640 pixels. Batch size was set to 64, initial learning rate was set to 0.01, and the learning rate was dynamically adjusted using the cosine annealing algorithm. The smoothing parameter was set to 0.1. To prevent overfitting, the weight decay was set to 0.0005, and the momentum parameter was set to 0.937. The model was iterated for 150 rounds. The IoU threshold was set to 0.45. During model training, the model parameters were continuously optimized to reduce the value of the loss function, resulting in an excellent performing model. The loss function curve was plotted during training to evaluate the training results, with the batch as the *x*-axis and the loss value as the *y*-axis, showing the convergence of the model during the training process. As shown in Fig. 5, $box_{loss}$ represents the localization loss, $obj_{loss}$ represents the confidence loss, and $cls_{loss}$ represents the classification loss. In the first 33 rounds, the localization loss on the training set was relatively high, reaching above 0.06, and it decreased to 0.045 by the 96th round. In the first 62 rounds, the confidence loss on the training set was relatively high, reaching above 0.17, and it decreased to 0.148 by the 140th round. In the first 66 rounds, the classification loss on the training set was relatively high, reaching above 0.01, and it decreased to 0.073 by the 114th round. As the model was trained, the loss values gradually decreased. There was noticeable fluctuation in the first 30 rounds. By observing the changes in each curve, the function eventually reached a converged state at the 150th iteration round.

### Performance evaluation of real-time head-up rate detection model

When detecting the head-up rate in the classroom, the accuracy of the detection is one of the key metrics to focus on. For head-up rate detection, it can be considered as a binary classification problem, with two tasks: head-up and head-down classification. In this study, we introduce the Confusion matrix^38^ to analyze the performance of the classification model. It visualizes the correspondence between the predicted results of the classification model and the true labels in the form of a table. For a binary classification problem, the Confusion Matrix is a $2 \times 2$ matrix, as shown in Table 2.

**Table 2:** Confusion matrix.

Predicted category	Real category	
	True head-up rate	True head-down
Predicted head-up	TP	FP
Predicted head-down	FN	TN

*True Positive (TP)* The number of positive examples that the model correctly predicts as positive, *False Positive (FP)* The number of negative examples that the model incorrectly predicts as positive, *False Negative (FN)* The number of positive examples that the model incorrectly predicts as negative, *True Negative (TN)* The number of negative examples that the model correctly predicts as negative.

**Figure 6:**
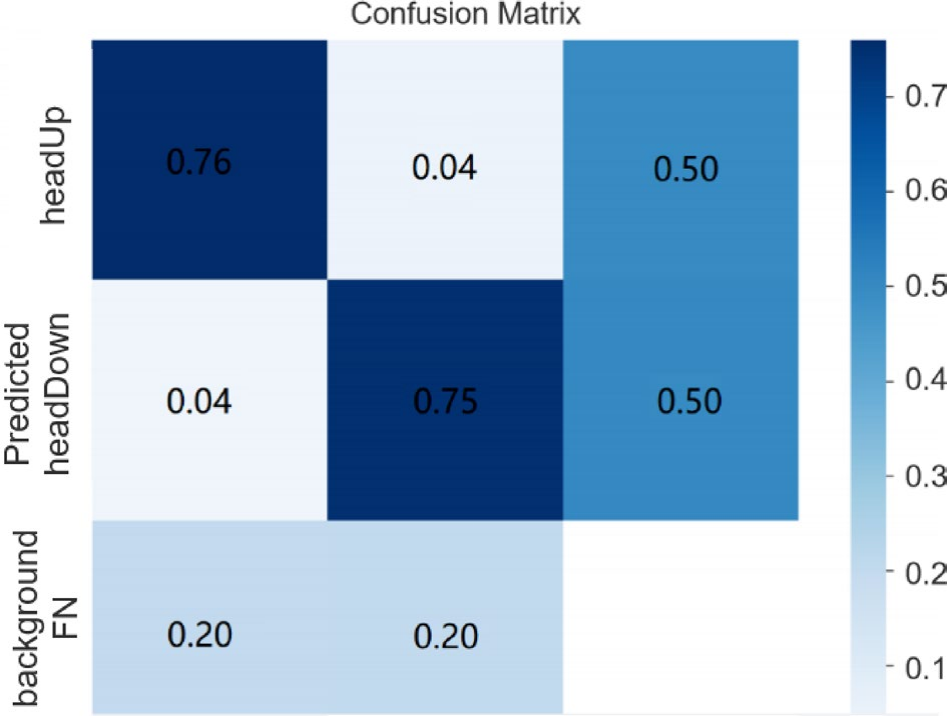
Confusion matrix after model training.

The confusion matrix obtained during model training in this study is shown in Fig. 6. The *x*-axis represents the true categories, from left to right: headUp, headDown, and background for false positives (FP). The *y*-axis represents the predicted categories, from top to bottom: headUp, headDown, and background for false negatives (FN). From Fig. 6, it can be observed that the probability of correctly predicting headUp as headUp is 0.76, the probability of correctly predicting headDown as headDown is 0.75, the probability of incorrectly predicting headDown as headUp is 0.04, and the probability of incorrectly predicting headUp as headDown is 0.04.

Precision and recall are important metrics for evaluating the performance of classification models. Precision represents the proportion of true positives among the samples predicted as positive, $Precision=TP/(TP+FP)$, while recall represents the proportion of true positives among the actual positive samples, $Recall=TP/(TP+FN)$. F1-score is the weighted average of precision and recall, which comprehensively evaluates the performance of the model in classification tasks. F1-score considers both precision and recall, making it a more comprehensive metric for evaluating the performance of the real-time head-up rate detection model.

11 $$\begin{aligned} F1{\text{-}}score=(2 \times (Precision \times Recall))/(Precision \times Recall). \end{aligned}$$

The F1-score provides a balanced measure of precision and recall. In the context of the real-time head-up detection model, it is crucial to consider both the ability to correctly identify true positives (precision) and capture all positives (recall). By using the F1-score, we can comprehensively evaluate the model’s performance while considering both precision and recall. This is particularly important in the classification task of distinguishing between students’ head-down and head-up behaviors. According to the results of the confusion matrix and Eq. (11), the real-time head-up detection model achieved an F1-score of 95%, indicating excellent performance in terms of precision and recall. The high F1-score suggests that the model exhibits outstanding performance in this classification task, indicating a high level of credibility and reliability. This reliability enables the model to effectively evaluate classroom teaching quality in practical applications, providing accurate reference and decision support for post-secondary education.

### Comparative analysis

#### Evaluation of classroom teaching quality based on survey questionnaire analysis

To evaluate the classroom teaching quality of a university in Shandong province, China, we utilize the Analytic Hierarchy Process (AHP)^39^ for statistical analysis of the survey questionnaire based on the indicator system of classroom teaching quality evaluation (as shown in Table 3).

**Table 3:** Indicator system for evaluating classroom teaching quality at a university in Shandong province.

Primary indicator	Secondary indicator	Tertiary indicator (Classroom observation point)
Classroom teaching quality	(1) Teaching skills	(1) The lecture relates theory to practice, with appropriate and vivid examples, and well-designed problems
		(2) The teacher has a proficient grasp of course content and practical operation skills.
	(2) Teaching attitude	(3) The teacher is enthusiastic and able to deliver the lecture without reading from notes
	(3) Professional ethics	(4) There is frequent communication between the teacher and students, with guidance provided on both academic and moral matters
		(5) The teacher shows concern for and provides guidance to students who have difficulty learning
		(6) The teacher listens to and responds to feedback from students
	(4) Teaching methods	(7) The teacher’s teaching methods promote ideological and political education
	(5) Teaching content	(8) The teacher’s emphasis, language, and clarity of organization during lectures, and the quality of multimedia courseware are good
		(9) The teacher is fully prepared for classes and has a proficient understanding of course content
	(6) Teaching organization	(10) The teacher’s classroom organization and management skills are good
	(7) Classroom situation	(11) Homework assignments and grading are done in a timely manner, with guidance and assistance provided to students
	(8) Student engagement	(12) Students exhibit good order during lectures or practical activities

The steps for evaluating the classroom teaching quality in the university using the Analytic Hierarchy Process (AHP) include: establishing a hierarchical structure, constructing judgment matrices, calculating weights, conducting consistency tests, and conducting comprehensive evaluations. First, determine the evaluation objectives, criteria, and sub-criteria. Then, construct judgment matrices through pairwise comparisons to assess the relative importance of each criterion. Next, calculate the weights and conduct consistency tests to ensure the rationality and consistency of the judgment matrices. Finally, based on the weights of the criteria, conduct a comprehensive evaluation of the quality of classroom teaching to obtain the final evaluation result.

#### Results comparative analysis

This study analyzed the classroom survey questionnaire data from 12 teachers using the Analytic Hierarchy Process (AHP) to obtain the evaluation scores for each classroom. Based on research conducted in universities, the results of the classroom teaching quality evaluation based on the survey questionnaire analysis were classified into five levels: Excellent (scores above 90), Good (scores between 80 and 90), Average (scores between 70 and 80), Fair (scores between 60 and 70), and Poor (scores below 60). According to this standard, the evaluation level for the classroom teaching quality analyzed through the survey questionnaire was “Excellent” for all teachers. For each of these teachers, real-time detection of the students’ head-up rate was conducted, and the detection results were normalized for the purpose of classroom teaching quality evaluation. It was found that the evaluation levels for two of the classrooms were “Poor” and “Average” respectively, as shown in Table 4.

**Table 4:** Comparative analysis of classroom teaching quality evaluation results.

No.	Classroom teaching quality evaluation results based on real-time head-up rate detection (Result A)			Classroom teaching quality evaluation results based on survey questionnaire analysis (Result B)	
	Classroom average head-up rate	Normalized real-time detection results	Classroom teaching quality level	Survey questionnaire analysis results	Classroom teaching quality level
1	0.6525	0.8661	Good	0.9534	Excellent
2	0.493	0.6544	Fair	0.906	Excellent
3	0.7534	1	Excellent	0.9635	Excellent
4	0.6213	0.8247	Good	0.9389	Excellent
5	0.6825	0.9059	Excellent	0.9512	Excellent
6	0.6423	0.8525	Good	0.9489	Excellent
7	0.6389	0.8480	Good	0.9498	Excellent
8	0.6931	0.9200	Excellent	0.9613	Excellent
9	0.6847	0.9088	Excellent	0.9594	Excellent
10	0.6745	0.8953	Good	0.9523	Excellent
11	0.6689	0.8878	Good	0.947	Excellent
12	0.5352	0.7104	Average	0.9213	Excellent

**Figure 7:**
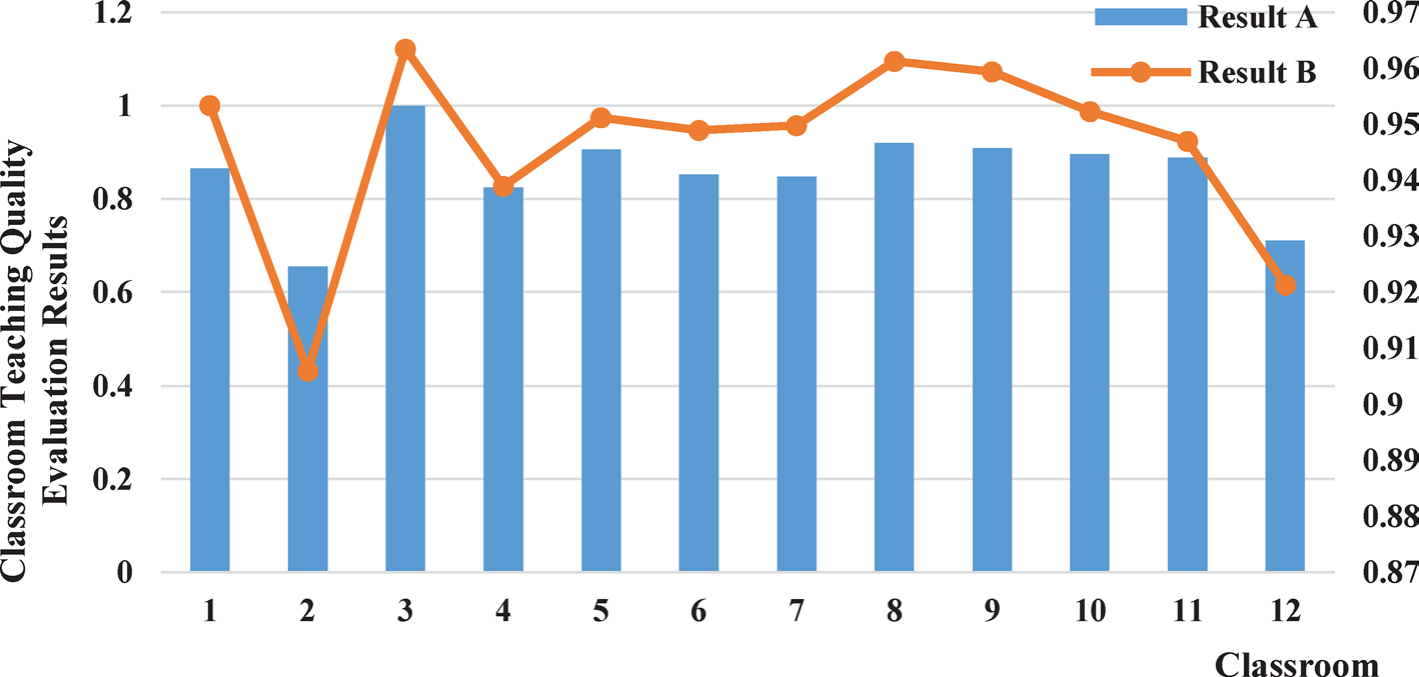
Visualization and comparative analysis of classroom teaching quality evaluation results.

Table 4 and Fig. 7 reveal that the overall patterns of classroom teaching quality evaluation results based on real-time head-up rate detection and survey questionnaire analysis are consistent. However, there are some discrepancies in the grading of the evaluation results. The survey questionnaire analysis method relies on students’ evaluation of the teacher’s classroom teaching effectiveness to determine the quality of classroom teaching. The evaluation results can be influenced by factors such as students’ personal preferences, course difficulty, and exam grades, leading to subjectivity and lack of objectivity. In comparison, the real-time head-up rate detection method assesses classroom teaching quality by monitoring students’ head-up rate in real-time, independent of students’ subjective awareness. This method provides more objective and accurate evaluation results, with a more distinct grading system. The more distinct grading results can offer more accurate and detailed information, aiding universities in improving educational quality and individual learning development. Furthermore, the real-time head-up rate detection method has the advantage of real-time evaluation. It can provide results in real-time during the class, helping teachers to promptly understand students’ engagement and make timely adjustments and feedback to enhance teaching effectiveness. In contrast, the survey questionnaire analysis method requires students to fill out questionnaires and undergo data analysis, resulting in some time delay.

In conclusion, the real-time head-up rate detection method for evaluating classroom teaching quality has significant advantages over the survey questionnaire analysis method in terms of objectivity and real-time evaluation. These advantages enable more accurate assessment of classroom teaching quality and assist universities in making timely teaching adjustments and improvements, thereby enhancing classroom teaching quality. Through the analysis and evaluation of actual teaching cases, this study found that small object detection technology can objectively and accurately evaluate classroom quality. It can monitor and analyze students’ behavior in real-time, identify issues, and identify areas for improvement. Moreover, small object detection technology can provide abundant data support for educational decision-making and personalized education.

## Conclusion

This paper proposes a real-time evaluation method for assessing the teaching quality in post-secondary classrooms based on the head-up rate. The method utilizes an improved YOLOv5 model for object detection to continuously monitor and detect the head-up rate of students in the classroom, thus evaluating the teaching quality. Experimental validation of our proposed model demonstrates its high accuracy, achieving a 95% F1 score. Comparative analysis further highlights the advantages of our real-time head-up rate detection method over traditional survey questionnaires. By using objective head-up rate data, our method reduces common issues of information inaccuracy and subjectivity in traditional evaluation methods. An important significance of our work is providing educators with an objective and real-time feedback mechanism to assess teaching quality. Through continuous monitoring and evaluation, our method enables timely adjustments and improvements in teaching effectiveness. This has the potential to enhance students’ overall learning experience and contribute to better educational outcomes. We acknowledge that there are challenges and limitations in our research. For example, the adaptability and accuracy of this method in specific environments need further investigation and improvement. Future research can explore the integration of other evaluation methods and indicators to establish a more comprehensive and multi-perspective system for assessing post-secondary classroom teaching quality.

## Data Availability

The data that support the findings of this study are available from Dean’s Office of Shandong University of Science and Technology but restrictions apply to the availability of these data, which were used under license for the current study, and so are not publicly available. Data are however available from Rui Wang (wangrui@sdust.edu.cn) upon reasonable request and with permission of Dean’s Office of Shandong University of Science and Technology.
